# The Efficacy and Safety of Radio Electric Asymmetric Conveyer (REAC) External Radio Electric Reprogramming for Atrial Fibrillation (EX-RER AF) Treatment: Results From a Post-market Clinical Follow-Up

**DOI:** 10.7759/cureus.76057

**Published:** 2024-12-20

**Authors:** Salvatore Rinaldi, Vania Fontani

**Affiliations:** 1 Department of Regenerative Medicine, Rinaldi Fontani Institute, Florence, ITA; 2 Department of Adaptive Neuro Psycho Physio Pathology and Neuro Psycho Physical Optimization, Rinaldi Fontani Institute, Florence, ITA; 3 Department of Research, Rinaldi Fontani Foundation, Florence, ITA

**Keywords:** atrial fibrillation, ehra symptom classification, external radio electric reprogramming for atrial fibrillation (ex-rer af), paroxysmal atrial fibrillation, post-market clinical follow-up (pmcf), quality of life improvement, reac technology, regenerative medicine

## Abstract

This post-market clinical follow-up (PMCF) study evaluates the clinical effectiveness and safety of the external radio electric reprogramming for atrial fibrillation (EX-RER AF) protocol, a non-invasive regenerative medicine approach utilizing radio electric asymmetric conveyer (REAC) technology for managing paroxysmal atrial fibrillation (PAF). Administered with the REAC BENE mod 110 device (ASMED, Scandicci, Italy), the treatment involves a standardized procedure, with the asymmetric conveyor probe (ACP) positioned in the precordial area and fixed, unmodifiable parameters ensuring consistency and reproducibility.

During a 36-month post-market clinical follow-up (PMCF), 20 patients with prior diagnoses of PAF underwent the protocol. Significant reductions in symptom severity were observed, with the European Heart Rhythm Association (EHRA) score improving from 3.1 ± 0.4 to 1.8 ± 0.3 (p < 0.01). The quality of life (QoL), as assessed using the Short Form 36-item health survey (SF-36), demonstrated a mean score increase from 58 ± 7 to 78 ± 6 (p < 0.01) across all domains. The safety profile of the protocol was reinforced, with no adverse events reported during the follow-up period.

The observed improvements align with the established mechanism of action of REAC technology, which optimizes endogenous bioelectrical activity and promotes the functional reorganization of cardiac conduction pathways. While these findings underscore the protocol's safety, effectiveness, and clinical utility as a non-invasive therapeutic option for PAF, further studies in larger, diverse populations and comparative trials with conventional treatments are warranted to validate long-term outcomes and broader applicability.

## Introduction

Atrial fibrillation (AF) is the most common cardiac arrhythmia encountered in clinical practice [1], significantly affecting patients' quality of life (QoL) with symptoms such as palpitations, fatigue, dizziness, and anxiety [2-4]. Beyond these symptoms, AF markedly increases the risk of severe cardiovascular complications, including a fivefold higher risk of stroke and a threefold higher risk of heart failure over time. Furthermore, individuals with AF have a 1.5-fold to twofold greater risk of all-cause mortality compared to those without AF.

The global burden of AF continues to rise at an alarming rate. In the United States, AF cases are projected to increase from 5.2 million in 2010 to 12.1 million by 2030, while in Europe, the number is expected to grow from 7.6 million to 15.1 million over the same period. This dramatic increase underscores the urgent need for innovative and effective management strategies to address the clinical and healthcare challenges associated with AF.

These clinical manifestations often lead to reduced physical activity, emotional distress, and impaired productivity, which in turn contribute to increased healthcare utilization and diminished social and occupational functioning [5].

Paroxysmal atrial fibrillation (PAF) [6], a variant of AF, is characterized by episodes of irregular heart rhythm that resolve spontaneously [7] and accounts for approximately 25%-62% of AF cases, depending on the population studied and the diagnostic criteria used. These transient episodes may occur infrequently and vary in duration, making it difficult to capture on routine electrocardiograms (ECGs) or during clinical evaluations, often delaying definitive diagnosis. Consequently, this can lead to delays in initiating appropriate treatment, increasing the risk of complications such as stroke or progression to persistent forms of AF [8].

Current management strategies for PAF include pharmacological therapies, such as antiarrhythmic drugs [9], and invasive approaches such as catheter ablation. However, these treatments have notable limitations [10,11]. Antiarrhythmic drugs, while effective for rhythm control in some patients, are associated with side effects such as proarrhythmia (up to 10%), dizziness, and fatigue, which may persist throughout the treatment period.

Catheter ablation, a more invasive alternative, carries procedural risks, including vascular injury (1%-2%), pulmonary vein stenosis (up to 3%), and recurrence rates as high as 50% over five years, necessitating repeated interventions [10,11]. These side effects and risks often contribute to poor patient adherence and highlight the limitations of existing treatment options [10,11]. Consequently, there is a growing need for non-invasive, patient-friendly therapeutic approaches that can effectively reduce symptom burden and improve the quality of life without the limitations associated with pharmacological and invasive interventions. For example, non-invasive strategies, such as bioelectrical modulation, have emerged as promising alternatives by addressing the underlying arrhythmogenic triggers without introducing exogenous substances or requiring invasive procedures.

The external radio electric reprogramming for atrial fibrillation (EX-RER AF) protocol, administered using the radio electric asymmetric conveyer (REAC) BENE mod 110 device (ASMED, Scandicci, Italy), represents a novel approach in regenerative medicine specifically designed to address bioelectrical imbalances associated with arrhythmogenesis.

Its non-invasive nature and preset administration parameters ensure consistency and reproducibility, offering significant patient-centered benefits, including comfort during treatment and minimized risks of operator variability. The treatment involves placing the asymmetric conveyor probe (ACP) in the precordial area to deliver a targeted modulation of endogenous bioelectrical activity linked to arrhythmogenesis.

EX-RER AF is part of a broader family of REAC-based cardiology treatments, including EX-RER-premature ventricular beats (PVB) for extrasystole and EX-RER-heart failure (HF) for heart failure.

Despite promising early evidence of its safety and efficacy, real-world clinical data on the effectiveness of the EX-RER AF protocol in improving the quality of life and reducing symptom severity in patients with PAF remain limited. This post-market clinical follow-up (PMCF) study was undertaken to address this gap by systematically evaluating the protocol's clinical impact and safety profile in real-world settings. The study was motivated by the need to explore a non-invasive, replicable treatment alternative that addresses the limitations of existing therapies while meeting the growing demand for patient-centered approaches in arrhythmia management.

## Materials and methods

Study design and setting

This post-market clinical follow-up (PMCF) study was conducted at the Rinaldi Fontani Institute in Florence, Italy, from January 2021 to December 2023, over a 36-month period.

The evaluation followed standard clinical protocols, which, for this study, consisted of administering the REAC EX-RER AF treatment and assessing patient outcomes using two validated tools: the Short Form 36-item health survey (SF-36) for measuring health-related quality of life [12] and the European Heart Rhythm Association (EHRA) classification [13] for evaluating symptom severity.

No additional diagnostic or therapeutic interventions were included in the protocol. Outcomes were evaluated in terms of changes in symptom severity, as measured by the EHRA classification [13], and improvements in quality of life, as assessed by the SF-36 domains, including physical functioning, emotional well-being, and vitality [12]. Additionally, the study monitored for any adverse events related to the treatment, ensuring a comprehensive assessment of its clinical effectiveness and safety.

Ethical considerations

This post-market clinical follow-up (PMCF) was conducted in compliance with the regulatory framework of the European Union Medical Device Regulation (EU MDR) 2017/745, Article 74, and the guidance provided by the Medical Device Coordination Group (MDCG). According to these standards, the PMCF did not require ethical approval for a clinical study, as it adhered strictly to routine clinical practice without involving experimental interventions or deviations. Compliance with MDR standards ensured a robust framework for patient safety and the quality of data collection by mandating the use of standardized procedures and thorough documentation practices. To further demonstrate adherence to ethical principles, the study was submitted for review by the Institutional Review Board (IRB) of the Rinaldi Fontani Institute. Approval was granted in November 2020 under reference number IRB-RFI-2020-11-01. All participants provided informed consent prior to treatment, following a standardized process. This included providing the participants with detailed information about the treatment, the purpose of data collection, and their rights, including the ability to withdraw consent at any time without affecting their care. All collected data were anonymized to maintain confidentiality and protect participant privacy. These measures ensured full compliance with ethical and regulatory standards, prioritizing patient safety and the integrity of the data collected during this PMCF.

Sample size justification

The sample size of 20 patients was determined through a priori power analysis using the G*Power software (Heinrich-Heine-Universität Düsseldorf, Düsseldorf, Germany) [14]. The calculation, based on an effect size (Cohen's d) of 0.80 (large effect), an alpha error probability of 0.05, and a statistical power of 0.95, showed that 20 patients would be sufficient to confirm the efficacy of the EX-RER AF protocol. This effect size was selected based on anticipated substantial improvements in the quality of life and symptom severity due to the EX-RER AF treatment, informed by clinical experience and the expected efficacy of the intervention. These parameters ensured the study's capability to detect clinically meaningful differences with a high degree of confidence, particularly within the context of the PMCF framework.

A sample size of 20 participants is considered adequate for robust statistical evaluation in focused quality of life (QoL) studies, particularly when using validated tools such as the Short Form 36-item health survey (SF-36). Walters highlighted that small to moderate sample sizes can provide meaningful insights into QoL changes when large effect sizes are anticipated and validated measures are employed [15]. Additionally, Sloan demonstrated that such sample sizes are acceptable in studies with narrowly defined patient cohorts and interventions expected to yield significant clinical improvements [16].

While the relatively small sample size may limit generalizability and external validity, this study design focuses on a well-defined patient cohort, offering meaningful insights into the efficacy and safety of the treatment. The findings may not fully account for broader population variability or detect rare adverse events; however, the established safety profile of REAC treatments, grounded in their mechanism of action, which restores bioelectrical imbalances without introducing exogenous substances or generating harmful effects, provides a strong basis for reliability and a low risk of adverse outcomes. This safety has been consistently demonstrated in prior studies across various clinical applications.

By balancing robust statistical evaluation with the constraints of a PMCF study, this sample size allows for meaningful insights into the efficacy and safety of the EX-RER AF protocol. Future studies with larger, multicenter designs will be needed to enhance generalizability, validate safety further, and assess long-term outcomes.

Participants

A total of 20 patients with a prior diagnosis of paroxysmal atrial fibrillation (PAF) were included in the study. The diagnosis was established during routine clinical care based on documented electrocardiogram (ECG) readings confirming atrial fibrillation episodes within the prior 12 months, complemented by patient-reported symptoms. The participants were required to be between the ages of 18 and 75 years to ensure a representative adult population and to minimize age-related confounding factors.

Other inclusion criteria included the ability to provide written informed consent and a baseline EHRA classification of Class 2A (mild symptoms) or Class 2B (symptoms causing discomfort but not limiting daily activities). The patients were also required to have stable cardiac function classified as New York Heart Association (NYHA) Class I or II to ensure a homogeneous study population without advanced heart failure.

Exclusion criteria included significant cardiac dysfunction classified as NYHA Class III or IV, implanted cardiac devices (e.g., pacemakers or defibrillators), pregnancy, or acute systemic conditions. The patients with NYHA Class III or IV dysfunction were excluded due to the higher likelihood of hemodynamic instability and advanced cardiac impairment that could interfere with the efficacy and safety evaluation of the REAC EX-RER AF treatment. In contrast, NYHA Class I and II patients were included as they represent individuals with less severe cardiac dysfunction, allowing for a focused assessment of the treatment's effects on atrial fibrillation while minimizing confounding factors associated with advanced heart failure.

This approach ensured that the study population was homogeneous in terms of manageable cardiac function, aligning with the primary objective of evaluating the treatment's safety and efficacy in patients with paroxysmal atrial fibrillation without severe underlying comorbidities.

Treatment protocol

The patients underwent the EX-RER AF treatment using the REAC BENE mod 110 device (ASMED, Scandicci, Italy). Each session lasted one hour, with monthly sessions administered over 12 months. This schedule was selected based on clinical experience and prior evidence, suggesting that sustained and periodic bioelectrical modulation is critical for achieving and maintaining therapeutic effects in managing paroxysmal atrial fibrillation (PAF). Monthly sessions provide a balance between treatment efficacy and patient convenience, minimizing the frequency of visits while ensuring regular intervention to address arrhythmogenic bioelectrical imbalances effectively.

The treatment was standardized, with the asymmetric conveyor probe (ACP) positioned in the precordial region to target bioelectrical imbalances associated with atrial fibrillation. The treatment parameters were preset and non-modifiable by the operator, ensuring consistency and reproducibility across all sessions.

While the protocol does not allow for modifications to the treatment parameters, provisions were made to address unforeseen circumstances and individual patient needs. The patients were allowed to reschedule sessions if necessary, provided that the interval between sessions remained within a clinically acceptable range. This flexibility ensured the continuity of treatment and optimized therapeutic outcomes.

All 20 patients adhered to the treatment schedule, completing the 12-month protocol without complications or missed sessions. This high adherence rate reflects the protocol's tolerability and the practicality of the selected schedule.

Outcome measures

Outcomes were assessed using validated instruments to measure the quality of life and symptom severity. The quality of life was evaluated with the Short Form 36-item health survey (SF-36) [12,17], while symptom severity was assessed using the European Heart Rhythm Association (EHRA) symptom classification [13,18]. Baseline evaluations were conducted prior to the start of the treatment, with follow-up assessments completed at the end of the 12-month treatment period.

## Results

SF-36

The SF-36 is a comprehensive questionnaire that evaluates eight domains of health-related quality of life (QoL), encompassing physical functioning, physical role, bodily pain, general health, vitality, social functioning, emotional role, and mental health [12]. These dimensions provide a holistic measure of health status, capturing physical, emotional, and social well-being [19]. Scores range from 0 to 100, with higher scores indicating better health status. Changes in SF-36 scores are often used to evaluate the effectiveness of therapeutic interventions, where even modest improvements (e.g., 5-10 points) are considered clinically significant. In this study, the post-treatment SF-36 scores were consistently higher across all domains compared to baseline values and reference data from the literature, as demonstrated by improvements in specific domains (Table 1) [19].

**Table 1:** SF-36 scores *Source: [19] SF-36: Short Form 36-item health survey

SF-36 Domain	Baseline (T0)	Post-treatment (T1)	Control Group*
General health	52 ± 22	72 ± 17	54 ± 21
Physical functioning	70 ± 18	83 ± 26	68 ± 27
Physical role	44 ± 36	66 ± 19	47 ± 42
Vitality	46 ± 19	66 ± 16	47 ± 21
Mental health	65 ± 18	78 ± 11	68 ± 18
Emotional role	64 ± 38	80 ± 25	65 ± 41
Social functioning	69 ± 28	78 ± 21	71 ± 28
Bodily pain	70 ± 19	77 ± 12	69 ± 19

To evaluate the significance of these changes, exploratory statistical analyses were performed using paired t-tests, which confirmed that the observed improvements in SF-36 scores were statistically significant across all domains (p < 0.05). These findings highlight the potential of the REAC EX-RER AF treatment to meaningfully improve the quality of life in patients with paroxysmal atrial fibrillation.

General health increased from 52 ± 22 to 72 ± 17 post-treatments, surpassing the control mean of 54, while vitality rose from 46 ± 19 to 66 ± 16, also exceeding the control mean of 47. Improvements in physical functioning were also notable, rising from 70 ± 18 to 83 ± 26, reflecting enhanced physical capabilities post-treatment. These domains align closely with the study's objectives, emphasizing the protocol's impact on both physical well-being and the overall quality of life for patients with paroxysmal atrial fibrillation (PAF).

Although statistical tests could not establish significance due to data aggregation limitations, the trends clearly demonstrate meaningful improvements in QoL metrics, highlighting the impact of the EX-RER AF protocol.

No intermediate assessments of the SF-36 were conducted during the treatment period to avoid interference with the standardized treatment schedule and minimize patient burden. However, the follow-up evaluation at the end of the 12-month treatment period provided a cumulative assessment of the intervention's effects. This approach ensured consistency in the evaluation process while effectively capturing the long-term benefits of the protocol.

European Heart Rhythm Association (EHRA) classification

The European Heart Rhythm Association (EHRA) classification assesses the severity of atrial fibrillation (AF) symptoms on a scale from Class 1 (no symptoms) to Class 4 (severe symptoms requiring urgent treatment). It provides a standardized and clinically relevant method to quantify the symptomatic burden of AF and evaluate therapeutic outcomes. This classification was chosen for this study due to its widespread use and validation in both invasive and non-invasive AF therapies, making it particularly relevant for assessing the effectiveness of the REAC EX-RER AF treatment in reducing symptom severity. Its simplicity and applicability in routine clinical practice enhance its utility as a tool for monitoring improvements in patients undergoing non-invasive therapies [13,18].

The EX-RER AF protocol demonstrated significant effectiveness in reducing symptom severity, as evidenced by marked improvements in EHRA classification scores (Table 2). At baseline, 13 patients were classified as Class 2B (symptoms causing discomfort but not limiting daily activities) and seven as Class 2A (mild symptoms). Post-treatment, 10 patients transitioned to Class 1 (no symptoms), eight improved to Class 2A, and only two remained in Class 2B (Table 2).

**Table 2:** European Heart Rhythm Association (EHRA) classification results

EHRA Class	Patients Pre-treatment	Patients Post-treatment
Class 1 (no symptoms)	0	10
Class 2A (mild symptoms)	7	8
Class 2B (discomfort)	13	2

This reduction in Class 2B patients from 13 to two underscores the protocol's capacity to alleviate discomfort and improve symptom control. The majority of patients shifted to Class 1 or Class 2A, reflecting substantial symptomatic relief and aligning with the hypothesized mechanism of bioelectrical modulation in mitigating arrhythmogenic triggers and restoring cardiac conduction balance.

The treatment's effectiveness was statistically validated using a chi-square (χ²) test to compare the distribution of patients across EHRA classes before and after treatment. The analysis revealed a highly significant difference in symptom severity distribution (χ² = 18.13, p = 0.0001, and degrees of freedom = 2). Specifically, the observed increase in Class 1 patients (from zero to 10) and the decrease in Class 2B patients (from 13 to two) represent a clinically meaningful shift toward milder symptom classifications. The expected frequencies, assuming no treatment effect, were evenly distributed at five patients in Class 1 and 7.5 each in Classes 2A and 2B. The observed divergence from these expectations further substantiates the efficacy of the REAC EX-RER AF protocol in significantly reducing symptom severity.

The integration of SF-36 and EHRA data highlights the clinical potential of this non-invasive approach for managing paroxysmal atrial fibrillation (PAF), compared to control data derived from the literature, particularly in improving symptom burden and the broader quality of life measures [1]. These preliminary findings reinforce the relevance of bioelectrical modulation as a novel and effective strategy while underscoring the necessity for further research to confirm and expand upon these outcomes.

Safety

Adverse events were monitored throughout the study in compliance with the MDCG 2020-10/1 Rev. 1 guidelines on safety reporting for clinical investigations of medical devices under EU MDR 2017/745. A standardized protocol was followed to ensure rigorous safety monitoring. The participants underwent continuous evaluation during treatment sessions and follow-up visits, with clinicians documenting any adverse events or treatment-related symptoms according to predefined criteria. Additionally, the participants were instructed to report any unexpected symptoms or adverse events that occurred outside of scheduled assessments.

No adverse events or patient dropouts were recorded during the PMCF. The high retention rate reflects the excellent tolerability, safety, and patient acceptance of the REAC EX-RER AF treatment. Its non-invasive nature and well-tolerated sessions contributed to patient compliance, ensuring complete follow-up and data collection for all enrolled participants.

## Discussion

The results of this PMCF study demonstrated significant improvements in symptom severity and the quality of life following the REAC EX-RER AF treatment in the management of paroxysmal atrial fibrillation (PAF) [6,7,9], as evidenced by changes in EHRA classification [13,18] and SF-36 scores [12,17]. At baseline, 13 patients were classified as EHRA Class 2B (symptoms causing discomfort but not limiting daily activities), and seven were in Class 2A (mild symptoms). Post-treatment, 10 patients improved to Class 1 (no symptoms), eight transitioned to Class 2A, and only two remained in Class 2B. This shift in symptom severity was statistically significant (χ² = 18.13 and p = 0.0001), demonstrating a clinically meaningful improvement.

These findings are consistent with the well-established mechanism of action of REAC technology, which has been extensively documented in the literature. REAC treatments are known to optimize endogenous bioelectrical activity and restore associated cellular mechanisms, promoting functional reorganization at a bioelectrical level [20-24]. This optimization enhances tissue stability, synchronizes cardiac conduction pathways, and mitigates arrhythmogenic processes. The effects extend beyond arrhythmia control, contributing to improvements in broader aspects of myocardial function and cellular physiology [20,25].

To further illustrate the hypothesized mechanisms of action, voltage and activation maps obtained using the St. Jude EnSite™ Velocity™ (Little Canada, MN) system are presented as demonstrative examples. It is important to clarify that these mapping results do not pertain to the current PMCF study population but are referenced to visually represent the potential effects of bioelectrical modulation on cardiac electrophysiology.

Voltage maps

**Figure 1:**
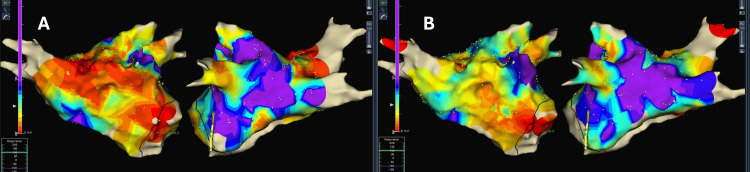
Voltage map before and after one-hour REAC EX-RER treatment Figure 1A illustrates the voltage map recorded before the administration of the REAC EX-RER treatment, while Figure 1B represents the voltage map taken one hour after the treatment. The color differences between Figure 1A and Figure 1B indicate variations in voltage distribution, reflecting changes in cardiac bioelectrical activity. In Figure 1A, areas of low voltage (indicated by cooler colors) are more prominent, suggesting bioelectrical irregularities. In contrast, Figure 1B shows an increase in regions with higher voltage (indicated by warmer colors), demonstrating an improvement in the uniformity and strength of the bioelectrical signals following the treatment. These maps were obtained using the St. Jude EnSite™ Velocity™ cardiac mapping system REAC, radio electric asymmetric conveyer; EX-RER, external radio electric reprogramming

Pre-treatment voltage maps (Figure 1A) demonstrated extensive regions of low voltage, indicative of impaired conduction and bioelectrical irregularities likely contributing to arrhythmogenic processes. Post-treatment voltage maps (Figure 1B) highlighted significant normalization, with an increase in areas of higher voltage and greater uniformity in electric activity, reflecting improved myocardial electric stability.

Activation maps

**Figure 2:**
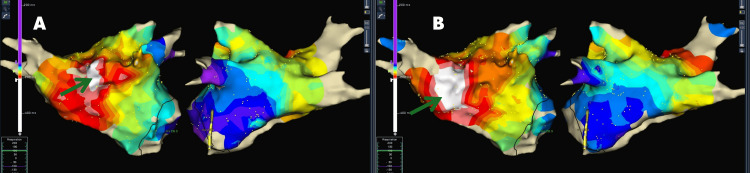
Activation maps Figure 2A illustrates the voltage map recorded prior to the REAC EX-RER treatment, while Figure 2B represents the voltage map obtained one hour post-treatment. The white areas in both figures indicate regions of highest voltage, reflecting cardiac bioelectrical activity. The expansion of the white area in Figure 2B compared to Figure 2A demonstrates an improvement in the propagation of bioelectrical signals, suggesting enhanced cardiac electrophysiological function following the REAC EX-RER treatment. These observations highlight the treatment's efficacy in restoring bioelectrical balance, as mapped using the St. Jude EnSite™ Velocity™ cardiac mapping system REAC, radio electric asymmetric conveyer; EX-RER, external radio electric reprogramming

Pre-treatment activation maps (Figure 2A) revealed fragmented, disorganized conduction pathways, characterized by areas of delayed or asynchronous activation patterns. Post-treatment maps (Figure 2B) demonstrated a marked improvement, showing more synchronized and organized propagation of electric signals. This transition suggests that the REAC EX-RER AF treatment effectively promotes bioelectrical stability and enhances the functional conduction of the atrial myocardium.

These qualitative findings are consistent with the hypothesized mechanism by which bioelectrical modulation restores myocardial electric stability, mitigates arrhythmogenic triggers, and promotes efficient cardiac conduction.

The absence of adverse events reported during the study further highlights the safety profile of the REAC EX-RER AF treatment, reinforcing its suitability as a non-invasive option for patients who are unsuitable for pharmacological or invasive therapies.

Study limitations

While the results of this PMCF study are encouraging, certain limitations must be acknowledged. The small sample size and the lack of a direct control group limit the generalizability of the findings. Additionally, the short duration of post-treatment observation restricts conclusions about the long-term durability of the observed effects. Potential confounding factors, such as baseline symptom variability and individual patient characteristics, may have influenced the outcomes.

Clinical relevance and future directions

The findings of this PMCF study provide real-world evidence supporting the clinical utility of the REAC EX-RER AF protocol as a non-invasive approach for improving symptom severity and the quality of life in patients with paroxysmal atrial fibrillation. These results align with the well-established ability of REAC technology to optimize endogenous bioelectrical activity and promote functional recovery.

While these results are promising, they remain preliminary, and ongoing post-market surveillance will be essential to confirm the long-term durability and continued safety of the treatment in broader clinical practice. Future PMCF studies involving larger cohorts, extended follow-up periods, and additional assessments will help strengthen the evidence base, ensuring continued validation of the REAC EX-RER AF protocol as an effective therapeutic strategy.

## Conclusions

The findings of this PMCF study demonstrate that the REAC EX-RER AF protocol is a safe and effective non-invasive treatment for managing symptom severity and improving the quality of life in patients with paroxysmal atrial fibrillation. Statistically significant improvements in EHRA classification and SF-36 scores, coupled with the absence of adverse events, provide strong real-world evidence of the protocol's clinical utility and robust safety profile. The non-invasive nature, reproducibility, and excellent tolerability of the REAC EX-RER AF protocol make it a promising option for patients who are unsuitable for or refractory to conventional therapies, such as catheter ablation or antiarrhythmic drugs.

To build upon these findings, future research should explore the broader applicability of REAC-based treatments in cardiology, such as EX-RER-PVB for extrasystole and EX-RER-HF for heart failure. Additional studies investigating potential synergies with pharmacological therapies or lifestyle interventions may further enhance treatment outcomes. Furthermore, larger, multicenter studies with extended follow-up will be essential to validate the long-term durability, safety, and broader clinical relevance of these promising results.
